# Two Cases of Group A Streptococcus-Induced Right Empyema: Rare Occurrences in Adult Medicine

**DOI:** 10.7759/cureus.68920

**Published:** 2024-09-08

**Authors:** Cheuk Cheung Derek Leung, Pak Yui Fong, Yu Hong Chan, Man Ying Ho, Yiu Cheong Yeung

**Affiliations:** 1 Medicine and Geriatrics, Princess Margaret Hospital, Hong Kong, HKG

**Keywords:** clindamycin, empyema, group a streptococcus, pleural infection, streptococcus pyogenes

## Abstract

Group A Streptococcus (GAS) empyema, though rare in adults, poses serious clinical challenges. We present two cases of GAS-induced right empyema in immunocompetent patients. Case 1 involved a 45-year-old female Chinese healthcare worker with persistent pleural effusion despite antibiotic therapy. GAS was isolated from her sputum and bronchoalveolar lavage, necessitating a treatment shift to clindamycin and co-amoxiclav. Case 2 featured a 55-year-old Filipino domestic helper exhibiting right lower chest consolidation and effusion. Thoracocentesis confirmed empyema, prompting intrapleural fibrinolytic administration. Both cases highlight the diagnostic complexity and therapeutic intricacies of adult GAS empyema, underscoring the importance of early recognition and tailored management strategies for optimal patient outcomes.

## Introduction

Streptococcus pyogenes, also known as group A Streptococcus (GAS), is a Gram-positive coccus that resides in the throat and on the skin. It spreads through respiratory droplets or direct contact with infected respiratory secretions. It is possible for individuals to carry GAS without experiencing any symptoms, while others may develop infections of varying severity [1]. GAS is mostly associated with skin infection, scarlet fever, or pharyngitis. Less commonly, it causes invasive GAS (iGAS) infection, defined as a positive culture in normally sterile sites, such as blood, pericardial, pleural, cerebrospinal, or joint fluid. Toxic shock syndrome may also occur as a serious complication [2]. While relatively common in children [3-6], GAS empyema has only been reported in several literatures in adults [2,7-15]. In this article, we report two cases of right-sided empyema due to GAS in two immunocompetent patients.

## Case presentation

Case 1

A 45-year-old Chinese female presented with a six-day history of right-sided pleuritic chest pain, fever, and cough. She was a non-smoker and non-drinker and worked in healthcare settings with no significant travel history. On admission, she was afebrile, had a blood pressure of 104/57 mmHg, and had a pulse rate of 72 beats per minute. Her oxygen saturation was 95% on 2 L of oxygen. Her cardiovascular and abdominal examination was unremarkable. Chest examination revealed reduced vesicular breath sounds to the right upper zone and right lower zone and reduced right-sided chest expansion. No skin wound was identified.

**Figure 1:**
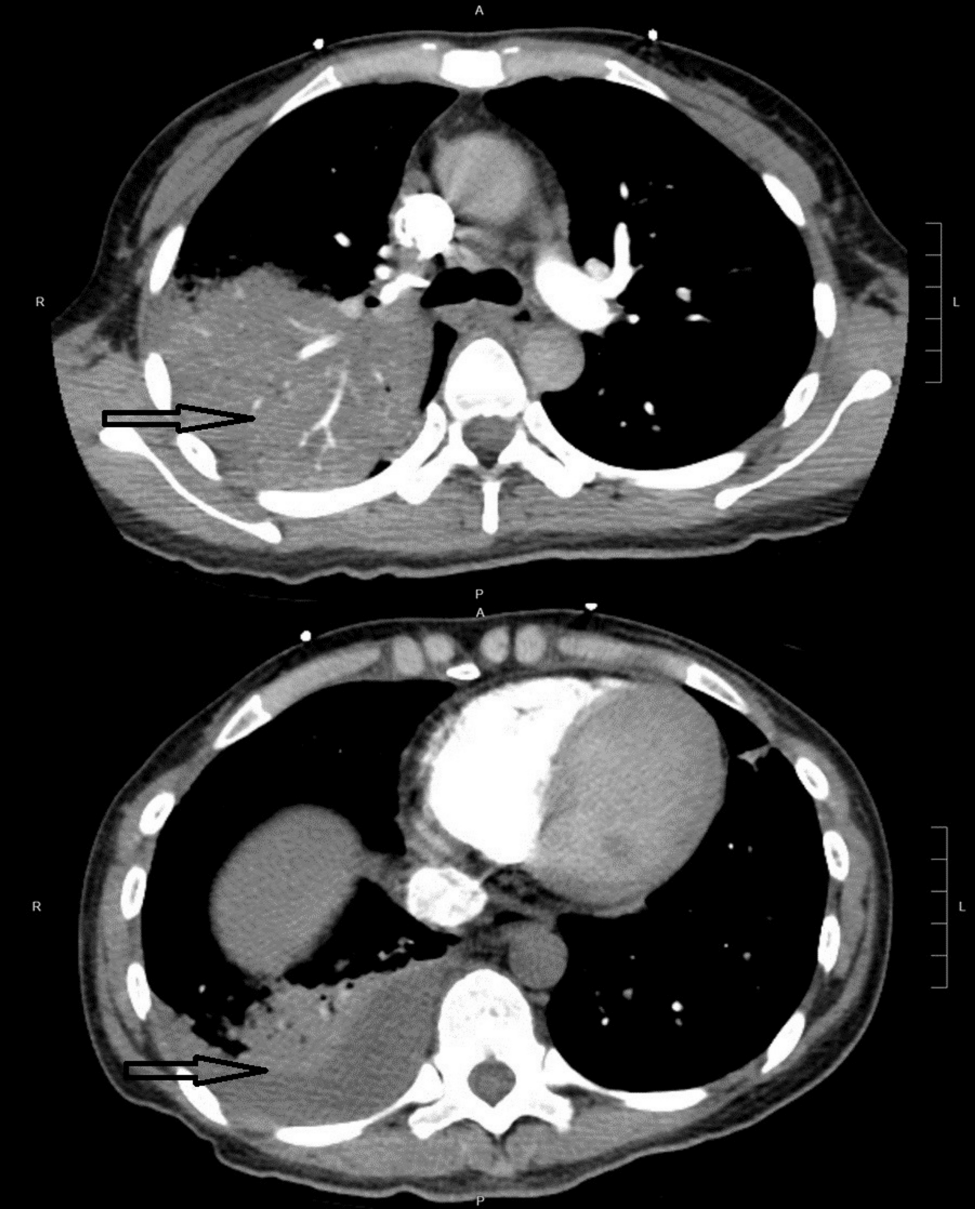
Computed tomography pulmonary angiogram images of Case 1 showing right upper lobe consolidation and a small right pleural effusion.

Her blood tests showed a raised white cell count of 13.3 x 10^9^/L, urea level of 13.2 mmol/L, and creatinine of 97 umol/L. Her anti-HIV antibody was negative; the liver function test was normal. Procalcitonin and C-reactive protein (CRP) were raised at 7.01 ng/mL and 253 mg/L, respectively. The electrocardiogram showed a sinus rhythm of 95 beats per minute. Nasal pharyngeal swab (NPS) reverse transcriptase polymerase chain reaction (RT-PCR) did not detect RNA of common respiratory viruses. Atypical pneumonia screening, including NPS for mycoplasma DNA, urine legionella antigen, and pneumococcal antigen, were negative. A computed tomography pulmonary angiogram (CTPA) (Figure 1) was performed. Florid consolidation was seen throughout the right upper lobe. Scattered multifocal ground glass and consolidations were also seen in both lungs. There were prominent mediastinal and bilateral hilar lymph nodes, less than 1 cm in diameter. A small right pleural effusion was present. Initial right-sided thoracocentesis yielded 2 mL of straw-colored pleural fluid only. Intravenous Ceftriaxone 1 g Q12H and oral doxycycline 100 mg Q12H were administered, but the clinical response was sub-optimal.

**Figure 2:**
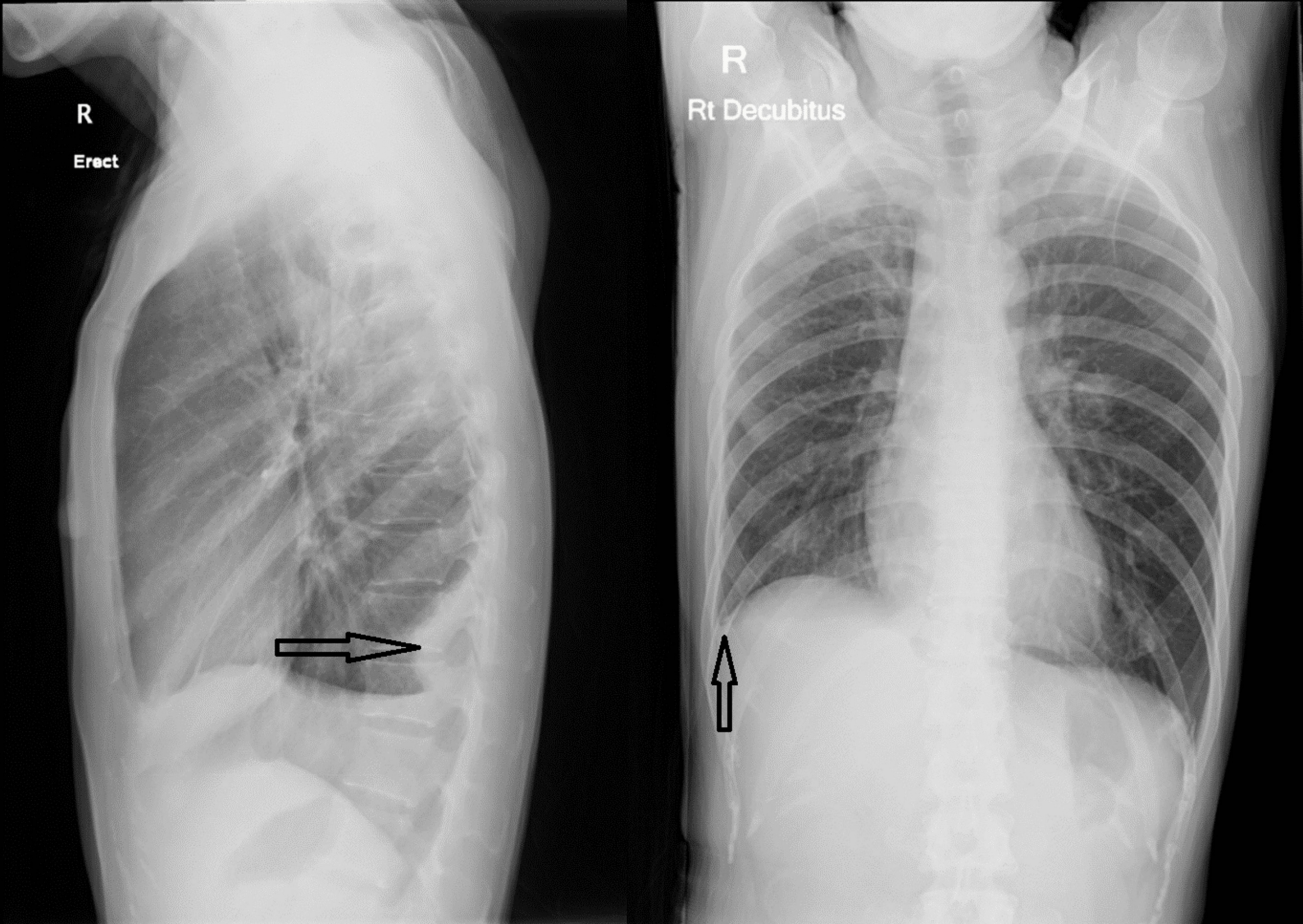
Right lateral and decubitus chest X-ray of Case 1 after treatment of group A Streptococcus empyema, showing resolved right upper lobe consolidation and a small loculated effusion in the posterior aspect of the right lower lobe. The cavitations in the right upper lobe have turned into thin-walled cysts, compatible with pneumatoceles.

A bronchoscopy was performed on the third day of admission and showed no endobronchial lesions. Bronchoalveolar lavage (BAL) was saved over the right upper lobe and eventually cultured GAS, sensitive to penicillin, clindamycin, and erythromycin. Acid-fast bacilli and fungal culture were negative. The sputum culture saved on admission also grew GAS. Antibiotics were changed to clindamycin 900 mg Q8H and co-amoxiclav 1.2 g Q8H. However, the patient’s inflammatory markers remain elevated, and serial chest x-rays showed an increased volume of right pleural effusion. On the sixth day of admission, right-sided thoracocentesis was repeated and yielded 600 mL of turbid fluid. Greater than 1,000/cubic mm of white blood cells were present in the pleural fluid, with a distribution of 40.9% polymorphs and 59.1% mononucleated cells. The pleural fluid was exudative, with a pH of 8.0, elevated protein of 33 g/L, adenosine deaminase (ADA) of 116 U/L, and lactate dehydrogenase (LDH) of 2,785 U/L. The pleural fluid culture was negative. Chest drain insertion was declined by the patient after discussing the risks and benefits. Follow-up chest x-rays showed interval reduction of right pleural fluid, and she was discharged with oral co-amoxiclav 1 g Q12H and oral clindamycin 450 mg Q8H. She received 17 days of intravenous antibiotics, followed by 11 days of oral antibiotics. One month after admission, her CRP normalized to <0.6 mg/L, and her chest x-ray showed resolved consolidation and a small loculated effusion in the posterior aspect of the right lower lobe. The cavitations previously noted have turned into thin-walled cysts, compatible with pneumatoceles (Figure 2).

Case 2

A 55-year-old Filipino domestic helper, with unremarkable past health and no travel history, presented to our unit for cough, shortness of breath, and right-sided chest discomfort for three days. Her temperature was 38 degrees Celsius on admission with a heart rate of 104 beats per minute. Her oxygen saturation was 94% on 2 L of oxygen. Physical examination revealed reduced air entry with crepitations over the right lower chest. Her initial blood tests revealed leukocytosis of 16.7 x 10^9^/L, urea level of 6.9 mmol/L, and creatinine level of 98 mmol/L. Her bilirubin and alanine transaminase (ALT) levels were elevated at 54 umol/L and 119 U/L, respectively. The alkaline phosphatase (ALP) level was normal. CRP and procalcitonin levels were both elevated at 291 mg/L and 4.1 ng/mL, respectively. Chest X-ray showed right lower zone consolidation and effusion occupying 1/3 of the right hemithorax. Blood culture showed no growth, while the sputum culture only yielded commensal growth. The laboratory test results of both cases are summarized in Table 1.

**Table 1:** Summary of laboratory findings of Case 1 and Case 2. WCC - White cell count; ALT - Alanine transaminase; ALP - Alkaline phosphatase; CRP - C-reactive protein; HIV - Human immunodeficiency virus; ADA - Adenosine deaminase; NPS - Nasal pharyngeal swab; RT-PCR - Reverse transcriptase polymerase chain reaction; rRNA - ribosomal RNA

Laboratory findings	Case 1	Case 2	Reference range
Blood tests			
WCC	13.3 x 10^9^/L	16.7 x 10^9^/L	3.7-9.2 x 10^9^/L
Urea	13.2 mmol/L	6.9 mmol/L	2.6 - 6.6 mmol/L
Creatinine	97 umol/L	98 umol/L	45 - 84 umol/L
Bilirubin	10 umol/L	54 umol/L	5 - 21 umol/L
ALT	13 U/L	119 U/L	< 35 U/L
ALP	103 U/L	115 U/L	30 - 120 U/L
CRP	253 mg/L	291 mg/L	< 5.0 mg/L
Procalcitonin	7.01 ng/mL	4.1 ng/mL	< 0.5 ng/mL
Anti-HIV Antibody	Negative	Negative	N/A
Pleural fluid analysis			
Gram stain and culture	Negative	Negative	N/A
Nanopore 16s rRNA gene sequencing	N/A	S.pyogenes	N/A
pH	8.0	6.8	N/A
WCC	>1000/cubic mm	>1000/cubic mm	N/A
Protein	33 g/L	52 g/L	N/A
LDH	2785 U/L	6085 U/L	N/A
ADA	116 U/L	81 U/L	< 27 U/L
Atypical pneumonia screening			
NPS RT-PCR of common respiratory virus	Negative	Negative	N/A
NPS for mycoplasma DNA	Negative	Negative	N/A
Urine legionella antigen	Negative	Negative	N/A
Urine pneumococcal antigen	Negative	Negative	N/A

**Figure 3:**
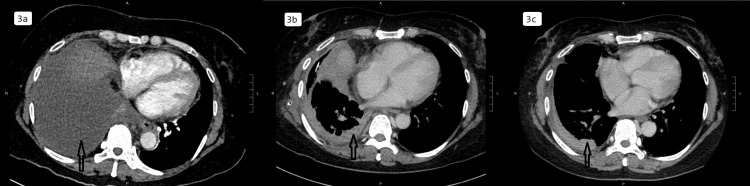
Computed tomography images of Case 2 before (3a), during (3b), and after treatment of group A Streptococcus (3c).

She was initially given intravenous co-amoxiclav 1.2 g Q8H and three days of oral azithromycin 500 mg daily, without good clinical nor radiological response; co-amoxiclav was therefore stepped up to intravenous piperacillin/tazobactam 4.5 g Q8H. A CTPA (Figure 3a) was performed, which showed gross right pleural effusion with right middle and lower lobe collapse, with no evidence of pulmonary embolism. Right-sided thoracocentesis four days after admission yielded 200 mL of turbid yellow fluid. Further analysis showed exudative pleural fluid with elevated white blood cells of >1,000/mm^3^ (48.3% polymorphs and 51.7% mononuclear cells), pH of 6.8, protein 52 g/L, LDH of 6,085 U/L, ADA of 81 U/L, and negative culture result, confirming the presence of a right-sided empyema. A right-sided intercostal chest drain was inserted two days later. The patient was continued on piperacillin/tazobactam with good clinical response; fever was downtrend, and oxygen was weaned off. Procalcitonin showed a downward trend to 0.31 ng/mL. However, there was poor output from the 12 French drain, and chest X-ray showed persistent right pleural effusion. Intrapleural fibrinolytics with alteplase 10 mg Q12H and dornase alpha 5 mg Q12H, for a total of six doses, were given via the chest drain. There was increased output from the drain afterwards, with a reduction of right pleural effusion on the chest X-ray. CT thorax 14 days after admission (Figure 3b) showed a significant reduction of the right pleural effusion. There were also two suspected small lung abscesses in the right upper and medial lobes. On the same day, nanopore 16s rRNA gene sequencing of pleural fluid revealed Streptococcus pyogenes. After discussion with the microbiologist, the antibiotic was switched to high-dose intravenous ceftriaxone 2 g Q12H. However, the patient developed a rash with ceftriaxone; thus, the antibiotic was switched to oral levofloxacin 500 mg daily, and she was subsequently discharged. She completed six days of ceftriaxone and 28 days of levofloxacin in total. Interval CT thorax (Figure 3c), four weeks after initiation of ceftriaxone, showed resolving right pleural collection and right lung abscesses with residual consolidative change. The patient remained afebrile. White cell count and CRP level were normalized at 5 x 10^9^/L and 1.4 mg/L, respectively.

## Discussion

Since December 2022, a rise in iGAS infections has been recorded in various countries across the globe, especially among children [16-21]. The incidence of GAS empyema in children was also reported to have increased significantly [3-5]. One pediatric study found GAS caused 73% of community-acquired pneumonia treated with chest tube drainage in 2022-2023 [5]. In adult medicine, GAS empyema remains extremely rare. A literature search found only 10 articles describing 15 cases of GAS empyema [2,7-15], with the earliest case diagnosed in 1987, reported by Barnham et al. [14]. The male-to-female ratio was 1:1 among individuals aged between 29 and 59 years old, with unremarkable past health. The mortality rate was 6.7%, with one patient dying from septic shock [10]. Our two cases involved middle-aged females with good past health, aligning with existing literature.

For case one, although the pleural fluid culture was negative, there was pus in the pleural fluid, and GAS was cultured from both her sputum and BAL of the right upper lobe, sufficient to reach the diagnosis of GAS empyema. For case two, the sputum and pleural fluid cultures were both negative, but GAS was identified with 16S rRNA gene sequencing of the pleural fluid sample. Pleural fluid culture in the setting of empyema has a low sensitivity rate of 18-60% [22,23] and a long incubation time of 24-72 hours [24]. 16S rRNA gene sequencing, a culture-independent microbiome study, has a significantly higher yield of the pathogen at 89% compared to 18% in controls with bacterial culture only in a case-controlled study on pleural fluid samples [25]. Preliminary results may be provided within six hours [26]. This diagnostic tool has gained popularity in the past few years, and future research is required to investigate its impact on patient outcomes and management. 

Once the diagnosis of GAS empyema was made, case 1 was treated with co-amoxiclav (a beta-lactam antibiotic) and clindamycin, while case 2 was treated with a beta-lactam antibiotic without clindamycin and later switched to levofloxacin (a fluoroquinolone antibiotic) due to skin rash. Both patients recovered well from their illness. Given the rarity of GAS empyema, there is no standardized antibiotic treatment. A combination of beta-lactam antibiotics together with clindamycin is generally recommended for iGAS infection [27]. The addition of clindamycin, a protein synthesis inhibitor with activity against toxin-producing streptococci, has been shown to improve the outcome of iGAS compared with beta-lactam monotherapy [28,29]. Levofloxacin is a reasonable alternative in the setting of penicillin allergy, with resistance rarely reported with GAS [30].

## Conclusions

In summary, we presented two rare cases of right-sided empyema caused by GAS in immunocompetent adults and discussed its epidemiology, diagnostic methods, and treatment options. The diverse clinical presentations and treatment responses in these immunocompetent individuals emphasize the need for a nuanced approach to managing such rare occurrences. Continued vigilance and prompt intervention are crucial in addressing GAS-induced empyema effectively, especially considering its atypical nature in adult populations. Further research and clinical awareness are warranted to enhance our understanding and management of this uncommon condition.
